# Triple-drug combination therapy versus six-month proton pump inhibitor monotherapy in non-*Helicobacter pylori Helicobacter* eradication, and hyperacid environment preference of *Helicobacter suis*: a clinical study

**DOI:** 10.1186/s12876-024-03252-5

**Published:** 2024-05-08

**Authors:** Toshihisa Tsukadaira, Seiichi Hayashi, Hiroyoshi Ota, Natsuko Kobayashi, Hiroyuki Agawa, Himiko Kodaira, Yasuhiro Sekiguchi, Takehisa Matsumoto, Kazuki Horiuchi, Tatsuya Negishi, Toshifumi Tada

**Affiliations:** 1 Department of Internal Medicine, Kenwakai Hospital, Kanaenakadaira, Iida, 1936, 395-0801 Japan; 2 Department of Pathology, Kenwakai Hospital, Kanaenakadaira, Iida, 1936, 395-0801 Japan; 3 Department of Biomedical Laboratory Sciences, Shinshu University School of Medicine, 3-1-1, Asahi, 390-8621 Matsumoto, Japan; 4 Department of Laboratory Sciences, Gumma University Graduate School of Health, 3-39-22 Showa-cho, Maebashi, 371-8511 Japan; 5 Department of Laboratory Medicine, Shinshu University Hospital, 3-1-1, Asahi, 390-8621 Matsumoto, Japan; 6 Department of Internal Medicine, Japanese Red Cross Society Himeji Hospital, 1-12-1 Shimoteno, 670-8540 Himeji, Japan

**Keywords:** Non*-Helicobacter pylori Helicobacter*, *Helicobacter suis*, Eradication, Triple-drug combination therapy, Proton pump inhibitor, Gender, Hyperacid environment.

## Abstract

**Background:**

At present, eradication regimens for non-*Helicobacter pylori Helicobacter* (NHPH) have not been established yet. We investigated effectiveness of the standard triple-drug combination therapy for *Helicobacter pylori* eradication and of a proton pump inhibitor (PPI) monotherapy in eradication of NHPH.

**Methods:**

Subjects were the patients who were diagnosed with NHPH-infected gastritis based on microscopic findings, helical-shaped organisms obviously larger than *Helicobacter pylori*, in the gastric mucosal specimens using Giemsa staining at Kenwakai Hospital between November 2010 and September 2021, whose NHPH species were identified by polymerase chain reaction (PCR) analysis of urease genes in endoscopically-biopsied samples, and who consented to NHPH eradication with either the triple-drug combination therapy for one week or a PPI monotherapy for six months. Six months after the completion of eradication, its result was determined with esophagogastroduodenoscopy, microscopic examination, and PCR analysis. In cases of unsuccessful eradication, a second eradication with the other therapy was suggested to the patient.

**Results:**

PCR analysis detected NHPH in 38 patients: 36 as *Helicobacter suis* and two as *Helicobacter heilmannii*/*Helicobacter ailurogastricus*. Fourteen *Helicobacter suis*-infected and one *Helicobacter heilmannii*/*Helicobacter ailurogastricus*-infected patients requested eradication therapy. The triple-drug combination therapy succeeded in four of five patients, while the PPI monotherapy succeeded in five of 10 patients. Three of five patients who had been unsuccessful with the latter therapy requested the triple-drug combination therapy as the second eradication and all three were successful. In total, the triple-drug combination therapy succeeded in seven out of eight (87.5%) attempted cases, while the PPI monotherapy in five out of 10 (50%) attempted cases.

**Conclusions:**

In NHPH eradication, the triple-drug combination therapy was considered to be effective to some extent and to become the first-line therapy. While, although less successful, PPI monotherapy appeared to be a potentially promising option particularly for patients with allergy or resistance to antibiotics. Effectiveness of PPI monotherapy may be attributed to hyperacid environment preference of *Helicobacter suis* and PPI’s acid-suppressive effect. Additionally, male predominance in NHPH-infected gastritis patients may be explained by gender difference in gastric acid secretory capacity. However, further evidence needs to be accumulated.

**Study registration:**

This study was approved by the Research Ethics Committee of Kenwakai Hospital (No. 2,017,024).

## Introduction

Non-*Helicobacter pylori Helicobacter* (NHPH) was first reported by Heilmann et al. [1] and Dent et al. [2] in 1987. NHPH has certain pathogenicity such as being involved in the development of gastric mucosa-associated lymphoid tissue (MALT) lymphoma [3–5]. In addition, although less pathogenic than *Helicobacter pylori* (*H. pylori*), NHPH has been reported to be a possible cause of gastric cancer [6]. Therefore, eradication therapy should be considered when a diagnosis as NHPH infection was made.

At present, eradication regimens for NHPH have not been established yet. There have not been many reports of NHPH eradication, and the regimen used in these reports is the three-drug combination therapy composed of amoxicillin 750 mg twice a day, clarithromycin 200 mg twice a day, and vonoprazan 20 mg twice a day for seven days. This is the same as the first-line regimen for *H. pylori* eradication.

In this study, subjecting NHPH-infected patients whose NHPH-bacterial species were identified by urease gene analysis, we investigated the effectiveness of not only this three-drug combination therapy for a week and but also a proton pump inhibitor (PPI) monotherapy for six months in NHPH eradication.

## Patients, methods, and ethical issue

### Including criteria of the patients

Patients who met all of the following criteria were subjected: the patients (1) who underwent esophagogastroduodenoscopy (EGD) at our facility, Kenwakai Hospital (Iida, Japan), between November 2010 and September 2021, (2) who underwent gastric mucosal biopsy, (3) who were diagnosed with NHPH infection by microscopic examination of biopsied specimen based on Giemsa staining, (4) who offered another biopsied sample for use in polymerase chain reaction (PCR) analysis with an additional advance informed consent, (5) in whom NHPH bacterial species was identified by PCR analysis, and (6) who consented to eradication therapy for NHPH infection after being briefed in detail on the pathogenicity and the eradication therapy of NHPH.

### Pathology

**Fig. 1:**
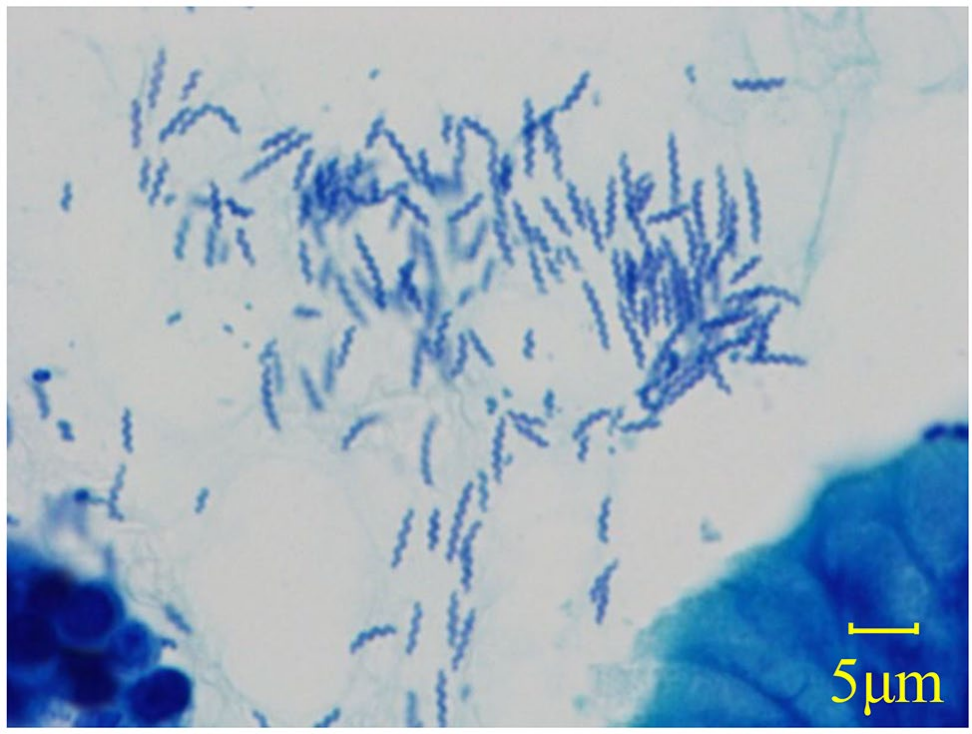
Pathological examination: NHPH is observed as relatively long and tightly coiled organisms by Giemsa staining (original magnification, ×1,000)

NHPH infection was diagnosed when a helical-shaped organism obviously larger than *H. pylori* was microscopically observed in the gastric mucosal specimen by using Giemsa staining [Fig. 1].

### PCR analysis

DNA isolation, PCR analysis and sequencing of urease A and B genes were as below. Gastric mucosal biopsy samples for PCR analysis from NHPH-positive patients were freshly frozen and stored in a deep freezer until analysis. Then, DNA was isolated from the freshly frozen samples with DNeasy Blood & Tissue Kit (Qiagen). All the extracted DNA was examined by urease PCR assay, and sequencing for identifying gastric NHPH as follows. PCR amplifications and sequencing of urease gene were done according to O’Rourke et al. [7]. Urease genes were amplified using primer pairs U430F and U1735R. PCR products were purified using the Fast Gene Gel/PCR Extraction Kit (Nippon Genetics). The products were directly sequenced with the same primers and other primers (U850F, U1050R) using a BigDye Terminator cycle sequencing ready reaction kit and a 3500 Genetic Analyzer (Thermo Fisher Scientific). The sequences were compared with those in the NCBI GenBank (accession no. AB968247) by using the BLAST search tool (http://blast.ncbi.nlm.nih.gov/Blast.cgi).

All the extracted DNA was examined by urease PCR assay, and sequencing for identifying gastric NHPH using primers listed in Table 1. PCR cycling conditions were as follows: the first-round PCR with primer sets U430F and U1735R consisted of initial denaturation at 95℃ for 2 min; 40 cycles of 94℃ for 30 s, 55℃ for 30 s, and 72℃ for 1.5 min; followed by a final extension step of 72℃ for 7 min. For the second reaction with the primer sets U430F and U1050R, or U850F and U1735R, the 100-fold dilution of the first PCR products were used as the template: initial denaturation at 95℃ for 2 min; 35 cycles of 94℃ for 30 s, 58℃ for 30 s, and 72℃ for 1.5 min; followed by a final extension step of 72℃ for 7 min.

**Table 1:** Primers used for urease PCR and sequencing reactions

Primer	Target gene	Purpose	Sequence (5’–3’)	Reference
U430F	Urease	PCR and sequencing	GCKGAWTTGATGCAAGAAGG	O’Rourke et al. [7]
U850F	Urease	PCR and sequencing	CAGCTGTGCGCTTTGAACCT	O’Rourke et al. [7]
U1050R	Urease	PCR and sequencing	TCTTCGCCATAAGTGGTGC	O’Rourke et al. [7]
U1735R	Urease	PCR and sequencing	CTTCGTGRATTTTAARRCCAAT	O’Rourke et al. [7]

Abbreviation: PCR, polymerase chain reaction.

O’Rourke et al. reported very high homology of urease genes within species or groups (> 97%), while homology between different species was low (73–83%) [7]. Although we did not set a clear cutoff for the identification of NHPH, 99–100% homology of the urease gene in strains identified as *Helicobacter suis* (*H. suis*) and 97–98% in strains identified as *Helicobacter heilmannii* (*H. heilmannii*)/*Helicobacter ailurogastricus* (*H. ailurogastricus*).

### NHPH-eradication therapy

Patients in whom NHPH species was identified by urease gene analysis were given a sufficient explanation of NHPH pathogenicity and eradication therapy, and then chose one of the following three options. Patients who requested and agreed to any of the eradication therapies were initiated on the therapy of their choice. Patients who did not requested or who requested but did not consent to any of the therapies were followed up without treatment.

1. Follow-up without positive treatment.
2. Eradication with the first-line treatment regimen for eradication of *H. pylori* in Japan (the standard triple-drug combination therapy, that is administration of amoxicillin 750 mg twice a day, clarithromycin 200 mg twice a day, and vonoprazan 20 mg twice a day for seven days) at own expense. Because of decreased susceptibility of *H. pylori* to clarithromycin, the need for antibiotic susceptibility testing has been advocated prior to *H. pylori* eradication [8]. Since testing antibiotic susceptibility of NHPH was, however, difficult at the time of this study, we applied this regimen whose effectiveness and safety were established, without susceptibility testing.
3. Eradication with long-term administration of a proton pump inhibitor (PPI) alone at own expense (actually, taking esomeprazole 20 mg once daily on an empty stomach for about 6 months).

We had experienced and reported two cases of NHPH gastritis in which NHPH was unexpectedly eradicated by long-term administration of PPI as treatment for coexisting reflux esophagitis, albeit in a Japanese paper [9]. As this PPI monotherapy was not covered by Japanese health insurance for NHPH eradication, we obtained the approval of the ethics committee of our facility for the option 3) in addition to the option 2).

### Determination of the success or failure of eradication

In every patient who chose the option 2) or 3), EGD, microscopic examination, and urease gene analysis were performed six months after the completion of eradication therapy to determine the success or failure of eradication.

### Choice for the second time in case of eradication failure

Patients who were not successful in the eradication therapy were once again asked to choose the other of the two aforementioned eradication therapies or follow-up without treatment.

## Results

### Patient background

Table 2 shows the patient background in this study. A total of 39,860 patients underwent EGD at Kenwakai Hospital between November 2010 and September 2021. Of these, 4,198 patients underwent gastric mucosal biopsy. Of these 4,198 patients, 68 consisting of 63 men and 5 women were diagnosed as NHPH infection by microscopic examination based on Giemsa staining. Patient ages at diagnosis ranged from 38 to 71 years, with a mean age of 54.3 years. Of the 68 patients diagnosed with NHPH infection, 40 offered us another gastric mucosal biopsied specimen for use in PCR analysis with an additional advance consent. Of these 40 patients, NHPH was detected in 38 (95%). NHPH bacterial species was identified as *H. suis* in 36 patients and as *Helicobacter heilmannii* (*H. heilmannii*)/*Helicobacter ailurogastricus* (*H. ailurogastricus*) in two patients. Urease gene was not detected in the remaining two patients.

**Table 2:** Patient background of the study

Numbers of patients		
	(1) who underwent esophagogastroduodenoscopy:	39,860
	(2) who underwent gastric mucosal biopsy:	4,198
	(3) who were diagnosed with NHPH infection based on Giemsa staining:	68 (63 men and 5 women)
	(4) who offered another biopsy sample for use in PCR analysis:	40
	(5) in whom NHPH bacterial species was identified by PCR analysis:	38
	(6) who received eradication of NHPH as positive therapy:	15

Abbreviations: NHPH, non-*Helicobacter pylori Helicobacter*; PCR, polymerase chain reaction.

### Patients’ choices and results of eradication therapy

After given a sufficient explanation on NHPH pathogenicity and eradication therapy, 22 out of 36 patients infected with *H. suis* and one out of two patients infected with *H. heilmannii*/*H. ailurogastricus* chose the option 1) from the three aforementioned options, that is follow-up without positive treatment. The remaining 15 patients consisting of 14 infected with *H. suis* and one infected with *H. heilmannii*/*H. ailurogastricus* selected positive treatment with the option 2) or 3) as the first eradication therapy. These 15 patients had no history of *H. pylori* or NHPH eradication.

Table 3 shows our examination on the NHPH eradication. Of the 14 *H. suis*-infected patients who requested positive treatment, five chose the option 2), with successful eradication in four and failure in one. The one patient in failure chose follow-up without another eradication. While, the remaining nine of the 14 *H. suis*-infected patients who requested positive treatment chose the option 3), with successful eradication in four and failure in five. Of the five patients in failure, two chose follow-up without another eradication. The remaining three chose the option 2) as the second eradication, and all were successful in eradication. One *H. heilmannii*/*H. ailurogastricus*-infected patient who requested positive treatment chose the option 3) and was successful in eradication. For convenience, counting each eradication therapy performed with the option 2) or 3) as one case, the number of attempted eradication cases amounted 18.

**Table 3:** NHPH species of the patients who requested eradication therapy, each patient’s choice and its result

Patient No.	Age	Sex	NHPH species identified by PCR analysis of urease genes	Choice for the first time and its result		Choice for the second time and its result	
				Choice	Result	Choice	Result
1	46	M	*Helicobacter suis*	Triple therapy	Success		
2	42	M	*Helicobacter suis*	PPI monotherapy	Failure	Follow-up	
3	40	M	*Helicobacter suis*	PPI monotherapy	Success		
4	55	M	*Helicobacter suis*	Triple therapy	Success		
5	62	M	*Helicobacter suis*	PPI monotherapy	Success		
6	45	M	*Helicobacter suis*	PPI monotherapy	Failure	Triple therapy	Success
7	62	M	*Helicobacter suis*	PPI monotherapy	Failure	Triple therapy	Success
8	61	M	*Helicobacter suis*	PPI monotherapy	Success		
9	68	M	*Helicobacter suis*	PPI monotherapy	Failure	Follow-up	
10	41	M	*Helicobacter suis*	PPI monotherapy	Failure	Triple therapy	Success
11	55	M	*Helicobacter suis*	Triple therapy	Success		
12	54	M	*Helicobacter suis*	Triple therapy	Success		
13	44	M	*Helicobacter suis*	PPI monotherapy	Success		
14	60	M	*Helicobacter suis*	Triple therapy	Failure	Follow-up	
15	55	M	*Helicobacter* *heilmannii/Helicobacter ailurogastricus*	PPI monotherapy	Success		

### Success rates of each eradication therapy

In total, eradication with the option 2), the triple-drug combination therapy, that is the first-line treatment regimen for *H. pylori* eradication in Japan, succeeded in seven out of the eight attempted cases (success rate: 87.5%). On the other hand, eradication with the option 3), long-term administration of a PPI alone, succeeded in five out of the 10 attempted cases (success rate: 50%). In any of the 18 cases, no discrepancies were observed between the results of microscopic examination and urease gene analysis which were performed 6 months after the completion of eradication therapy for determining the success or failure of eradication. In other words, the existence of NHPH was confirmed also by urease gene analysis when detected by microscopy, and not by urease gene analysis when not detected by microscopy.

### Case of unsuccessful eradication with the triple-drug combination therapy

In the only one case in which eradication with the triple-drug combination therapy failed, the patient, a 60-year-old man, was naturally healthy, had no underlying medical conditions, and underwent an EGD for the purpose of a health check-up. He had good adherence to his medication.

### Cases of follow-up without treatment

Since NHPH has been considered to be involved in the development of gastric MALT lymphoma, we instructed all patients who had chosen the option 1), follow-up without treatment, to continue undergoing EGD once a year.

All 22 *H. suis*-infected patients who had chosen the option 1), follow-up without treatment, underwent EGD one year after the choice of treatment or not. Further, four among the 22 underwent EGD once again one more year after that. One *H. heilmannii*/*H. ailurogastricus*-infected patient who had chosen the option 1) underwent an EGD one year after the choice of treatment or not. During these following-up EGDs, no samples were collected for the purpose of microscopic examination or PCR analysis. In all 23 patients in total who had chosen the option 1), no improvement in gastritis in the gastric antrum was endoscopically observed. In addition, the development of gastric MALT lymphoma was not observed in any of these following-up EGDs in any of the patients.

None of the patients have changed their requests and undergone eradication therapy.

## Discussion

### Pathogenicity of NHPH

NHPH causes infection in the human gastric mucosa, induces gastritis, and is associated with gastric MALT lymphoma. Although acute gastritis and acute gastric mucosal lesions (AGML) caused by NHPH have been reported in previous studies [10–12], most NHPH infections also cause chronic gastritis without symptoms [13]. Regarding MALT lymphoma, Stolte et al. found it in seven of 202 patients with NHPH gastritis [3], and Okiyama et al. found it in four of 15 patients with NHPH gastritis [5]. In addition, Yasuda et al. reported that NHPH may be a possible cause of gastric cancer although less pathogenic than *H. pylori* [6]. Therefore, eradication therapy is recommended for patients diagnosed with NHPH infection.

### Gender of NHPH-infected patients

Regarding the gender of those infected with NHPH, men accounted for 73% of 111 cases and 80% of 15 cases in the reports of Stolte, et al. [14] and Okiyama, et al. [5], respectively. Similarly in the present study, male was predominant (63 of 68 patients; 92.6%). This male predominance in NHPH infection, not seeming to have been adequately discussed in the past, may be explained by gender difference in gastric acid secretory capacity. Gastric acid secretory capacity is reported to be higher in men than in women [15, 16]. As discussed below, because NHPH prefers acidic environments, NHPH infection may occur more commonly in men than women.

### Species of NHPH

As to NHPH bacterial species in Japanese patients, Øverby et al. reported that they confirmed *H. suis* and *H. heilmannii s.s.* in seven and one cases out of 12 cases, respectively, and did not identify the species in the remaining four cases [17]. Similarly in the present study, *H. suis* was predominant among the cases in which the species was identified (36 of 38 patients; 94.7%).

### Effectiveness of the triple-drug combination therapy for eradication of NHPH

For the purpose of NHPH eradication, a regimen originally for *H. pylori* eradication have been used in Japan. Administration of a proton pump inhibitor, amoxicillin, and clarithromycin for seven days is considered the first-line treatment for NHPH as well as *H. pylori* infection, as the standard triple-drug combination therapy. In previous case reports on Japanese patients, eradication with this regimen was successful in all cases [18–20]. In addition, a multicenter study in Japan reported that NHPH eradication was attempted in 45 cases using this first-line regimen and in success in all cases [21]. However, at our facility, NHPH eradication with the triple-drug combination therapy failed in one out of eight attempted cases. It should be noted that the first-line regimen for *H. pylori* eradication may not be always effective, when proposing this regimen to patients.

### Effectiveness of PPI monotherapy for eradication of NHPH

On the other hand, the effectiveness of PPI monotherapy is also worth considering as a regimen for NHPH eradication other than the first-line regimen with three drugs.

We previously reported two cases of NHPH-infected gastritis in which NHPH were unexpectedly eradicated by long-term administration of a single PPI as treatment for coexisting reflux esophagitis [9]. In another study of ours in which we examined 50 cases of NHPH, regular arrangement of collecting venules (RAC) in the gastric corpus was observed in all cases, gastroesophageal reflux disease (GERD) of grade A or higher was observed in 45 cases, and gastric epithelial metaplasia of the duodenal bulb was observed in 38 cases [13]. Based on these endoscopic findings, we have mentioned that NHPH tends to infect hyperacid stomachs [13].

A report by Rimbara et al. reinforces our opinion. They reported that *H. suis* is more active in an acidic environment, and that the species does not grow and does suffer considerable damage in near neutrality [20]. Furthermore, Nakamura, et al. recently reported as below [22]. Many NHPHs showed the spiral form when the culture pH was 3.0. Ruthenium red *en bloc* staining during electron microscopy revealed that the coccoid form bacilli were observed in addition to the intact form at a pH of 5.0. The urease activity was the highest at a pH of 4.0, followed by that at a pH of 3.0. The motility of the bacteria was decreased at pH values of >5.0, compared to that at pH of 3.0 and 4.0. The aforementioned gender difference in NHPH infection may be explained by this acidic-environment preference of NHPH and the higher secretory capacity of gastric acid in men than women.

In the present study, we administered a PPI alone for six months to patients with NHPH-infected gastritis with the approval of our hospital’s ethics committee and successful eradication was confirmed in five of 10 attempted cases.

Based on the above, we consider that PPI monotherapy may be effective to some extent in eradicating NHPH, albeit with a lower success rate than the three-drug combination therapy. We believe that PPI monotherapy is worth trying for NHPH eradication because the risk of drug allergy and resistance is less than with the first-line treatment regimen that includes antibiotics. On the other hand, as the inhibitory effect of PPIs on NHPH may decrease when NHPH is in cocoid form, further study is needed on the type of PPI and the duration of PPI administration in order to improve the eradication effect.

### Limitation of the present study

Because the present study was retrospectively conducted at a single facility and with a small number of patients. it is difficult to conclude that a PPI monotherapy is effective.

In our study, we used esomeprazole as PPI, because esomeprazole was the most acid-suppressive drug available at the time. The use of other drugs now available, i.e. vonoprazan, should be considered in the examination of PPI’s effectiveness. In addition, as Rimbara et al. have successfully cultured *H. suis* in vitro directly from human stomachs [20], future studies should be conducted on the drug susceptibility of *H. suis* to PPIs.

## Conclusions

We examined the effectiveness of NHPH eradication with the triple-drug combination therapy for a week and/or a PPI monotherapy for six months in 15 patients with NHPH gastritis in whom detected organism was *H. suis* except in one patient. The former therapy, the first-line treatment regimen for *H. pylori* eradication in Japan, was considered effective to some extent also in NHPH eradication. On the other hand, the latter therapy, although less successful, may have been effective in NHPH eradication and may become a promising option, especially for patients with allergy or resistance to antibiotics. Randomized controlled trials with the triple therapy are needed to more accurately assess the effectiveness of PPI monotherapy.

The effectiveness of PPI monotherapy in NHPH eradication may be attributed to hyperacid-environment preference of *H. suis*, the predominant NHPH species in this study, and acid-suppressive effects of PPIs. Additionally, male predominance in NHPH-infected gastritis patients may be explained by gender difference in gastric acid secretory capacity. However, further evidence needs to be accumulated, such as examining the susceptibility of *H. suis* and other NHPH species to PPIs.

## Data Availability

The datasets generated and/or analyzed during the current study are available in the NCBI GenBank (accession no. AB968247).

## Abbreviations

NHPH: Non-*Helicobacter pylori Helicobacter*
PPI: Proton pump inhibitor
PCR: Polymerase chain reaction
EGD: Esophagogastroduodenoscopy
*H. suis*: *Helicobacter suis*
*H. heilmannii*: *Helicobacter heilmannii*
*H. ailurogastricus*: *Helicobacter ailurogastricus*
